# Betulinic acid and obesity-related disorders

**DOI:** 10.3389/fphar.2025.1674713

**Published:** 2025-09-23

**Authors:** Lara Azevedo, Ricardo Ferraz, Mónica Vieira, Cristina Prudêncio, Sílvia Fernandes

**Affiliations:** ^1^ RISE-Health, Center for Translational Health and Medical Biotechnology Research (TBIO), ESS, Polytechnic of Porto, Porto, Portugal; ^2^ Área Técnico-Científica de Ciências Químicas e das Biomoléculas, ESS, Polytechnic of Porto, Porto, Portugal; ^3^ LAQV-REQUIMTE, Departamento de Química e Bioquímica, Faculdade de Ciências da Universidade do Porto, Porto, Portugal; ^4^ Área Técnico-Científica de Anatomia Patológica, Citológica e Tanatológica, ESS, Polytechnic of Porto, Porto, Portugal; ^5^ Center for Research on Health and Environment (CISA), ESS, Polytechnic of Porto, Porto, Portugal

**Keywords:** health, obesity, betulinic acid, cancer, apoptosis, disorders

## Abstract

The obesity epidemic is not just a health issue, it is increasingly driving a shift in the prevalence of chronic diseases, affecting 890 million adults and straining healthcare systems worldwide. Conditions such as type 2 diabetes mellitus, cardiovascular diseases, non-alcoholic fatty liver disease, and various cancer types are closely tied to this growing crisis. Betulinic acid has anti-inflammatory, antioxidant and anti-cancer properties and modulates key metabolic pathways such as NF-κB and AMPK signaling. This compound improves insulin sensitivity, reduces hepatic steatosis, mitigates the progression of atherosclerosis and fibrosis, and suppresses inflammatory responses, which are important in treating those obesity-related disorders. Additionally, betulinic acid use in cancer treatment has been explored due to its potential in angiogenesis and metastasis inhibition and promotion of apoptosis. This review spotlights the therapeutic potential of the natural compound betulinic acid in processes such as insulin sensitivity, glucose and lipid metabolism, adiposity, inflammation, oxidative stress, intestinal microbiota, and other mechanisms underlying different obesity-related disorders. Overall, besides strong therapeutic potential of betulinic acid, described limitations such as poor aqueous solubility, limited bioavailability, production and extraction have resulted in scarce clinical data making it premature to draw definitive conclusions regarding its application in clinical practice.

## Graphical Abstract

**Figure:**
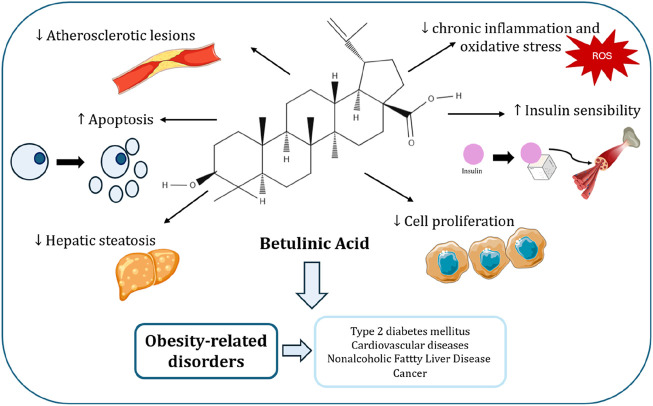
Image created with icons from vecteezy.com and smart.servier.com.

## 1 Introduction

Obesity is described as an excessive accumulation of body fat and is typically diagnosed when an individual’s body mass index (BMI) surpasses 30 kg/m^2^, according to the World Health Organization (WHO) (WHO. Obesity, 2025). Globally, obesity has been on the rise, affecting 890 million adults and significantly increasing the risk of mortality, highlighting the need for global preventive measures and effective interventions (WHO, 2025; Berrington de Gonzalez et al., 2010).

Obesity is related with multiple health issues, such as type 2 diabetes mellitus (T2DM), dyslipidemia, cardiovascular diseases (CVD), respiratory problems and many cancers, also called Obesity-Related Disorders (ORD) (Apovian, 2016). Furthermore, the interplay between genetic, environmental, and behavioural factors contributes to obesity, driven by greater food accessibility, reduced physical activity, and genetic predisposition (Singh et al., 2020). The treatment of obesity effectively involves several strategies, including dietary modifications, medication, such as Food and Drug Administration-approved drugs, and, in cases of severe obesity, bariatric surgery (Lemstra et al., 2016; Tak and Lee, 2020; Wiechert and Holzapfel, 2021).

**FIGURE 1:**
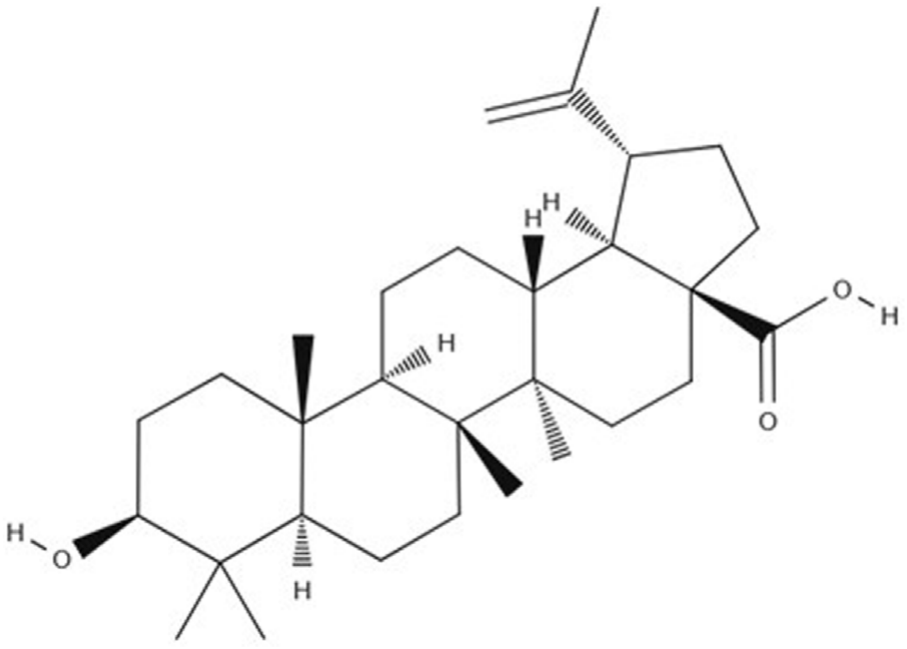
Betulinic Acid molecular structure.

As obesity becomes more prevalent and effective treatments increasingly difficult to achieve, there is a growing interest in alternative approaches, including natural bioactive compounds with the potential to address this health challenge. Betulinic Acid (BA) (Figure 1), a natural compound, has been demonstrated to prevent the accumulation of abdominal fat and promote weight loss, making it a promising candidate for use as a natural treatment for obesity (Zhu et al., 2024; de Melo et al., 2009).

This review aims to summarize the several ORD, reporting the mechanisms underlying these diseases, and explore the potential effect of BA in the treatment of these conditions.

## 2 Obesity-related disorders

### 2.1 Obesity and type 2 diabetes mellitus

T2DM occurs when the body cannot produce enough insulin or use the insulin that produces effectively, resulting in high levels of blood glucose (hyperglycemia) (Federation, 2025). In fact, some studies suggest that nearly 90% of T2DM occurrences are due to obesity (Pereira and Alvarez-Leite, 2014).

Insulin is fundamental for carbohydrates, proteins, and lipids regulation as it promotes glucose absorption from the bloodstream into adipose tissue, skeletal muscle cells, and liver (Wilcox, 2005). Insulin attaches to the α-subunits of its receptor, triggering the receptor’s autophosphorylation and the activation of insulin receptor substrates (IRS) (Tong et al., 2022; Zhang et al., 2020). This activates protein kinase B (PKB/Akt), which is essential for bringing glucose transporter 4 (GLUT4) to the cell membrane, enabling glucose to be absorbed into the cell (Tong et al., 2022; Zhang et al., 2020). Both obesity and T2DM are closely associated with insulin resistance, a condition where cells become less sensitive to the effects of insulin (Al-Goblan et al., 2014).

An increase in fatty acids (FA) from food intake and lipolysis of the adipose tissue leads to an elevated β-oxidation and a reduction in the use of glucose for metabolic processes, such as glucose intake and glycogen synthesis (Lois and Kumar, 2009; Shulman et al., 1990; Golay et al., 1984; Felber et al., 1987). This imbalance contributes to chronic hyperglycemia, impairing insulin sensitivity and eventually resulting in insulin resistance (Verma and Hussain, 2017). In T2DM, dysfunction in pancreatic β-cells significantly induces the progression of this disease, because these cells normally increase insulin production to compensate for rising blood glucose levels, especially in obesity, where insulin secretion is elevated and insulin clearance by the liver is reduced (Kahn et al., 2006; Kahn, 2001).

Prolonged exposure to high levels of glucose and FAs can damage β-cell function (Hauner, 2016). Chronic exposure to fatty acids leads to “glucolipotoxicity,” which impairs the β-cells’ ability to release insulin in response to glucose (Poitout and Robertson, 2008). This occurs through multiple pathways, including FAs binding to G-protein-coupled receptors on the β-cell membrane, which disrupts calcium balance and ultimately affects insulin secretion (Hauner, 2016; Itoh et al., 2003). Another mechanism involves the upregulation of uncoupling protein 2 (UCP-2), which interferes with the mitochondrial function essential for generating the ATP necessary for insulin secretion (Hauner, 2016; Chan et al., 2004). Therefore, defects in pancreatic β-cells function disrupt glycemic control, reducing insulin secretion and contributing to the progression of T2DM (Weyer et al., 1999; Gastaldelli et al., 2004).

Obesity also causes low-grade chronic inflammation, that is manifested by the activation of immune cells, which produces and releases pro-inflammatory cytokines such as tumour necrotic factor-α (TNF-α) and interleukin-6 (IL-6), and plays a major role in the development of T2DM (Chen et al., 2023). Those cytokines trigger the activation of c-JUN N-terminal kinase (JNK) and nuclear factor-kappa B (NF-κB) signalling pathways, the release of cytokines and chemokines, and the attraction of immune cells (Esser et al., 2014; Hirosumi et al., 2002). JNK and NF-κB pathways contribute to insulin resistance by facilitating the phosphorylation of serine and threonine residues on IRS, disrupting its interaction with the insulin receptor (IR), impairing downstream insulin signalling pathways and contributing to the progression of T2DM (Wu and Ballantyne, 2020).

Currently, one of the treatments for T2DM is metformin (Marín-Peñalver et al., 2016; Inzucchi et al., 2015). This compound enhances insulin sensitivity, reduces intestinal glucose absorption, and lowers hepatic glucose production by inhibiting gluconeogenesis in the liver and promoting glycolysis (Padhi et al., 2020; Quillen et al., 1999). However, this treatment has some side effects such as diarrhea, nausea, anorexia, and a decrease in the intestinal absorption of vitamin B12 (Marín-Peñalver et al., 2016). Therefore, new therapeutic approaches may be explored to provide better results for patients and resolve the challenges associated with current treatments.

### 2.2 Obesity and cardiovascular diseases

Obesity is closely linked to CVD such as coronary heart disease, hypertension, heart failure (HF), and ischemic stroke (Collaboration et al., 2011; Krauss et al., 1998; Kenchaiah et al., 2002). Obese patients have a twofold higher risk of developing HF relative to those with normal weight, highlighting a significant link between obesity and CVD (Kenchaiah et al., 2002). Moreover, obesity exacerbates dyslipidemia, insulin resistance, hypertension, and systemic inflammation, which are key risk factors (Ortega et al., 2016). The increase in body mass can significantly impact the cardiovascular system, leading to elevated blood volume, stroke volume, and cardiac output (Lavie et al., 2014). These changes result in higher filling volume and pressure, causing left ventricular dilation and hypertrophy, contributing to cardiac dysfunction (Lavie et al., 2014).

Adipose tissue dysfunction is strongly associated with obesity-related CVD. Excessive intake of high-calorie foods leads to adipocyte hypertrophy, lipid accumulation, and subsequent cell necrosis or apoptosis (Nakamura et al., 2014; Sun et al., 2010). This process recruits inflammatory cells and promotes the dysfunction of adipose tissue (Nakamura et al., 2014; Sun et al., 2010). Adipose tissue produces a range of adipokines such as cytokines (e.g., TNF-α, IL-6, IL-10), chemokines (e.g., MCP-1, CCL2, CCL5), acute-phase reactants (e.g., C-reactive protein (CRP)), damage-associated molecular pattern molecules (DAMPs), pro-inflammatory factors like leptin and anti-inflammatory factors like adiponectin (Landecho et al., 2019; Rodríguez et al., 2015). In the context of obesity, the secretion of these adipokines becomes dysregulated, establishing a link between excessive and dysfunctional adiposity and an elevated CVD risk (Landecho et al., 2019; Rodríguez et al., 2015).

Adiponectin and leptin, two key adipokines, play complex roles in the development of CVD. Low circulating adiponectin levels, commonly observed in obese patients, are associated with an increased risk of obesity-related CVD (Zhao et al., 2021). Similarly, leptin, which regulates food intake and energy balance, is elevated in obese patients and promotes low-grade inflammation and influences blood pressure by modulating sympathetic nervous activity, endothelial nitric oxide (NO) production, and vasoconstriction induced by angiotensin II (Landecho et al., 2019; Muruzáb et al., 2002; Gómez-Ambrosi et al., 2002; Rodríguez et al., 2010). However, obesity-induced leptin resistance leads to hyperleptinemia, a condition characterized by excessive leptin production, which contributes to vascular inflammation, insulin resistance, oxidative stress, and left ventricular hypertrophy, ultimately increasing CVD risk (Landecho et al., 2019; Kim et al., 2016; Wallace et al., 2001).

Obesity is also linked to elevated glucose levels and increased production of reactive oxygen species (ROS) and reactive nitrogen species by the endoplasmic reticulum (ER), which disrupts insulin secretion and sensitivity (Cercato and Fonseca, 2019; Keane et al., 2015). Insulin resistance promotes dyslipidemia, which is manifested by elevated levels of triglycerides, decreased levels of high-density lipoprotein (HDL), and the formation of small dense low-density lipoproteins (Ormazabal et al., 2018; Meshkani and Adeli, 2009; Robins et al., 2011). Dyslipidemia is also characterized by high levels of low-density lipoprotein cholesterol (LDL), which promote the formation of atherosclerotic plaques on arterial walls, contributing to atherosclerosis and cardiovascular complications (Alloubani et al., 2021; Elshourbagy et al., 2014). These interconnected mechanisms highlight the association between obesity and CVD, drawing attention to the urgent need for new therapeutic strategies to improve cardiovascular outcomes.

### 2.3 Obesity and nonalcoholic fatty liver disease

The hallmark of nonalcoholic fatty liver disease (NAFLD) is the presence of hepatic steatosis (≥5% of hepatocytes) without the evidence of other liver disease etiologies, such as viral hepatitis, the use of steatogenic medications, autoimmune hepatitis, Wilson’s disease, or excessive alcohol consumption (Younossi et al., 2016; Chalasani et al., 2012). NAFLD represents a major global health issue, affecting an estimated 30% of the population, with prevalence rates reaching as high as 90% among individuals with obesity (Amini-Salehi et al., 2024; Machado et al., 2006). Importantly, NAFLD can progress to more severe issues such as cirrhosis and hepatocellular carcinoma (Polyzos et al., 2019).

NAFLD is influenced by various factors, including genetic predispositions such as polymorphisms in the patatin-like phospholipase domain-containing protein 3 (PNPLA3) genes, dietary components (e.g., fructose), oxidative stress, adipokines, insulin resistance and gut microbiota dysbiosis (Polyzos et al., 2016; Valenti et al., 2010; Abdelmalek et al., 2010; Shanab et al., 2011; Jarrar et al., 2008; Sanyal et al., 2001). The diagnosis of NAFLD relies on the identification of hepatic steatosis through imaging techniques, including ultrasound, CT, MRI, MRS, or ultrasound-based transient elastography (FibroScan), or histological analysis (Sarwar et al., 2018; Brunner et al., 2019). Besides, blood-based biomarkers, such as elevated levels of aspartate aminotransferase levels (AST), alanine aminotransferase (ALT), and gamma-glutamyltransferase (GGT), may support the diagnosis (Brunner et al., 2019).

In obese individuals with NAFLD, lipoprotein lipase catalyzes the hydrolysis of circulating triglycerides into FA, which are then transported into hepatocytes by FA transport proteins (Perla et al., 2017; Petersen et al., 2007). This process is followed by an upregulation of *de novo* lipogenesis in the liver, resulting in excessive lipid deposition (Perla et al., 2017; Petersen et al., 2007). To attenuate this accumulation, the liver has two primary mechanisms for lipids elimination: FA oxidation, which occurs via β-oxidation and ketogenesis, and very-low-density lipoprotein (VLDL)-mediated export of triglycerides (Willis et al., 2021). Furthermore, *de novo* lipogenesis generates the intermediate metabolite, malonyl-CoA, that inhibits FA oxidation, promoting lipid accumulation and contributing to the pathogenesis of NAFLD (Willis et al., 2021; Mcgarry et al., 1977).

Another hallmark of NAFLD is the accumulation of triglycerides in hepatocytes as lipid droplets (Gonçalves et al., 2014). Oxidative stress, which arises from an imbalance between the production of ROS and the antioxidant defences, damages intracellular biological macromolecules, inducing cell dysfunction (Tong et al., 2022). This promotes the peroxidation of lipid and generates reactive aldehydes, amplifying hepatic inflammation by secretion of pro-inflammatory cytokines such as TNF-α, IL-6, and IL-1 (Delli Bovi et al., 2021; Day, 2002). In addition to mitochondrial dysfunction, ER stress further exacerbates ROS production, activates apoptotic pathways, and heightens lipotoxicity, contributing to chronic inflammation, liver fibrosis, and the progression of NAFLD to non-alcoholic steatohepatitis (NASH) (Delli Bovi et al., 2021).

Inflammation is a key factor in the transition of NAFLD into NASH (Du Plessis et al., 2015). This transition is driven by intrahepatic factors such as oxidative stress, mitochondrial dysfunction, and ER stress, leading to injury and hepatocellular metabolic dysfunction (Peiseler et al., 2022; Hardy et al., 2016). NAFLD inflammation is also regulated by extrahepatic factors such as gut-liver axis, adipose tissue inflammation, skeletal muscle, and bone marrow precursors (Peiseler et al., 2022; Nachit et al., 2021; Bäckhed et al., 2004). In obesity, adipose tissue undergoes macrophage activation, which promotes the production of pro-inflammatory mediators such as CRP and triggers inflammatory signalling pathways in the liver, including the secretion of TNF-α, IL-6 and IL-1β (Cordeiro et al., 2020; Weisberg et al., 2003; Guilherme et al., 2008). These cytokines, in turn, promote enhanced lipogenesis while suppressing FA oxidation, thereby establishing a connection between excessive lipid accumulation and an inflammatory status (Cordeiro et al., 2020; Guilherme et al., 2008; Kuzminova et al., 2016). Thus, the interaction between adipose tissue and the liver involves a network of inflammatory cells, adipokines, and cytokines, which are crucial in driving metabolic dysregulation and lipotoxicity (Cordeiro et al., 2020).

Dysbiosis of the intestinal microbiota, an imbalance frequently observed in obesity, emphasizes the role of the gut-liver axis in the NAFLD development (Porr et al., 2018; Karlsson et al., 2012). The gut-liver axis includes the intestinal epithelium, a crucial component of the gut barrier, which often exhibits dysfunction, known as “leaky gut” in individuals with NAFLD (Porr et al., 2018; Miele et al., 2009; Giorgio et al., 2014). This condition enhances gut permeability, contributing to an increase of toxins in the bloodstream, thereby promoting chronic low-grade inflammation, present in both obesity and NAFLD (Pezzino et al., 2022; Cani et al., 2009; Brun et al., 2007). Therefore, studies have demonstrated that in NAFLD patients, a correlation exists between increased gut permeability and the severity of hepatic steatosis (Cani et al., 2009; Brun et al., 2007).

Therapeutic approaches for NAFLD include the use of an anti-inflammatory agent named vitamin E, which has been found to improve the histological manifestations of NASH, although it does not significantly impact fibrosis severity (Said and Akhter, 2017). However, the development of additional therapeutic strategies remains essential to enhance outcomes for patients with NAFLD.

### 2.4 Obesity and cancer

The prevalence of cancer has been increasing over the years, with approximately 20 million new cases and 9.7 million cancer-related deaths reported worldwide in 2022 (Organization, 2024). Several risk factors have been associated with cancer, such as tobacco use, obesity, excessive alcohol consumption, a physically inactive routine and inadequate diet (Marino et al., 2024; Wu et al., 2018).

**FIGURE 2:**
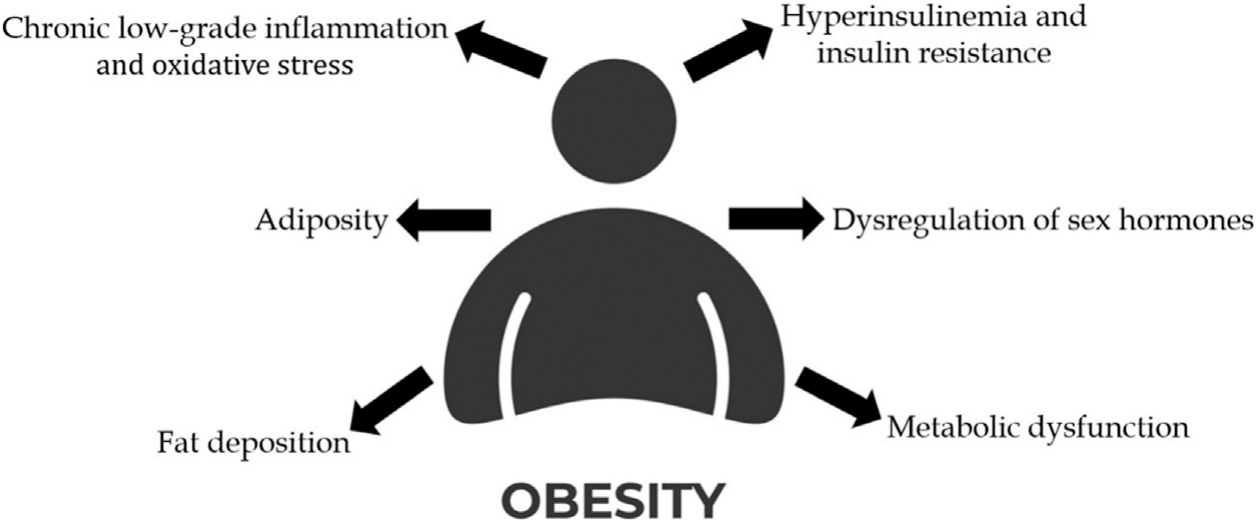
Representation of the mechanisms that link obesity to cancer development. (Created with an icon from vecteezy.com).

Notably, obesity is implicated in approximately 20% of all cancer cases, contributing to cancer development through several interconnected mechanisms, such as hyperinsulinemia and insulin resistance, dysregulation of sex hormones, chronic low-grade inflammation and oxidative stress, increased adiposity, the impact of ectopic fat deposition and metabolic dysfunction (Avgerinos et al., 2019; Karra et al., 2022; Wolin et al., 2010) (Figure 2).

Hyperinsulinemia, characterized by the increase of insulin levels due to the overproduction of insulin by pancreatic β-cells, promotes the production of insulin-like growth factor I (IGFI) (Poloz and Stambolic, 2015). This process is accompanied by reduced levels of IGF-binding proteins (IGFBP), which normally sequester IGFs in the liver (Poloz and Stambolic, 2015; Brismar et al., 1994). The resulting excess IGF and insulin activates multiple signalling pathways that promote cell proliferation, angiogenesis and inhibit apoptosis in malignant tissues (Xu et al., 2017; Tang et al., 2010; Teng et al., 2016).

Obesity induces a state of low‐grade chronic inflammation primarily driven by adipose tissue, which acts as a metabolically active organ secreting adipokines and pro-inflammatory cytokines with oncogenic potential (Booth et al., 2015). Cytokines such as IL-6, IL-1β, and TNF-α, increase the production of CRP and serum amyloid A, both of which are implicated in facilitating tumour progression (Friedenreich et al., 2021; Zhao et al., 2010; Visser et al., 1999). Oxidative stress represents another mechanism through which obesity influences cancer progression. ROS disrupts growth factors and mitogenic pathways, thereby promoting uncontrolled cell proliferation (DeNicola et al., 2011; Lee et al., 1998).

Obesity leads to increased adiposity which is implicated in cancer progression. Adipose tissue secretes leptin and adiponectin, two hormones with opposing functions. While adiponectin levels decrease in obesity, leptin levels rise, and the resulting leptin/adiponectin ratio can be associated with enhanced cancer aggressiveness (Ryan et al., 2003; Garofalo et al., 2006; Chen et al., 2006). Furthermore, the excess of adipose tissue increases aromatase activity, resulting in elevated estrogen levels, which result in increased risk of hormone receptor-positive breast and endometrial cancers (Avgerinos et al., 2019; Crosbie et al., 2010). Therefore, hormonal dysregulation and increased adiposity are key mechanisms underlying the association between obesity and cancer development.

Metabolic dysfunction is another mechanism stimulated by obesity that can promote cancer progression. *Warburg* et al. hypothesized that dysfunction in cellular respiration serves as the root cause of all cancers, where reduced respiration triggers tumorigenesis by increasing glycolysis to compensate for the energy loss deficit (Warburg, 1956). This increased glycolytic activity leads to the production of lactate by cancer cells under aerobic conditions–a phenomenon known as aerobic glycolysis - contributing to respiratory insufficiency that may become irreversible (Seyfried et al., 2014). Although aerobic glycolysis generates less ATP per molecule of glucose compared to oxidative phosphorylation (OXPHOS), its significantly higher rate compensates by enabling greater overall energy production, which is crucial for the proliferation of cancer cells (Vander Heiden et al., 2009).

The accumulation of body fat has been identified as a contributing factor in the development of several types of cancer, including postmenopausal breast, endometrial, colorectal, esophageal adenocarcinoma and prostate cancer, among others (Leitzmann et al., 2025).

Obesity significantly increases the risk of premenopausal triple-negative breast cancer by 67% and luminal B breast cancer by 73% compared to women of normal weight, according to information from the US Cancer and Steroid Hormone Study (Turkoz et al., 2013; Gaudet et al., 2011). Several obesity-driven mechanisms, such as hypoxia, increased levels of estrogens, insulin, adipokines, inflammatory mediators, and FFA, promote cancer cell growth (Brown, 2021). Adipose tissue hosts the cytochrome P450 enzyme aromatase, which converts adrenal-produced androgen precursors into estrogens, which can contribute to tumour progression by promoting DNA damage, stimulating cell proliferation, angiogenesis, and mutagenesis (Iyengar et al., 2013; Péqueux et al., 2012; Mobley and Brueggemeier, 2004; Zhao et al., 2006). Leptin, a hormone linked to breast cancer, activates the PI3K-AKT pathway, promoting cancer cell proliferation, epithelial-mesenchymal transition (EMT), and vascular endothelial growth factor (VEGF) expression, which enhances angiogenesis and invasion (Wei et al., 2016; Gonzalez-Perez et al., 2010). Increased levels of insulin and IGF-I, which promote cell proliferation and inhibit apoptosis in breast tissue, are two ways further ways that obesity contributes to this king of cancer (Chen et al., 2012; Weichhaus et al., 2012; Fortner et al., 2016). Additionally, obesity-induced chronic low-grade inflammation, which is characterized by enhanced macrophage infiltration and elevated expression of pro-inflammatory cytokines such as TNF-α and IL-6 in adipose tissue creates tumorigenic microenvironment (Weisberg et al., 2003).

Obesity is responsible for approximately 31% of cases of endometrial cancer, through mechanisms involving endogenous sex steroid hormones, inflammation, and insulin resistance (Pearson-Stuttard et al., 2018; Shaw et al., 2016). Notably, women with advanced-stage endometrial cancer (stage III and IV) and a BMI ≥40 face a significantly increased mortality risk compared to those with a normal weight, showing the critical need for effective anti-obesity interventions (Modesitt et al., 2007). Type I endometrial carcinomas manifest high estrogen levels and reduced progesterone levels (Shaw et al., 2016; Key and Pike, 1988). This imbalance arises from prolonged estrogen exposure without sufficient progesterone counteraction, leading to endometrial cell proliferation and increasing the risk of malignant transformation (Shaw et al., 2016; Key and Pike, 1988). Leptin, TNF-α, and IL-6, key factors associated with obesity, promote insulin resistance and stimulate the proliferation of endometrial cancer cells (Onstad et al., 2016). Additionally, chronic inflammation driven by obesity, enhances inflammatory cytokines levels, which promote cell proliferation and angiogenesis, consequently inducing endometrial cancer growth (Harvey et al., 2023).

Colorectal cancer is the third most prevalent cancer in the world, and its development is associated to obesity, as evidence by 2012 data showing that elevated BMI is responsible for 5.8% of new colorectal cancer cases in men and 7.0% in women (Pearson-Stuttard et al., 2018). Obesity drives inflammation through altered secretion of pro-inflammatory cytokines (e.g., L-6, TNF-α, PAI-1), adipokines, insulin, and IGF, alongside a reduction in adiponectin levels by adipose tissue (Ionescu et al., 2023). These inflammatory and hormonal changes allow the activation of signalling pathways that promote cancer cell proliferation and metastasis, thereby contributing to the development of colorectal cancer (Ionescu et al., 2023). Moreover, elevated levels of LPS, caused by gut microbiota dysbiosis, increase intestinal permeability and systemic endotoxemia, which promotes chronic inflammation that drives EMT, cell invasion, and metastasis, exacerbating colorectal cancer progression (Killeen et al., 2009; Liu et al., 2017). Additionally, bile acids secreted into the intestine induce inflammatory reactions, ROS production, epithelial damage, genomic instability, and apoptosis inhibition, collectively enhancing colorectal cancer progression (Ochsenkühn et al., 1999; Nguyen et al., 2018; Ye et al., 2020).

Esophageal adenocarcinoma (EAC) is also associated to obesity, being shown that individuals with a higher BMI face a twofold higher risk of developing EAC (Hoyo et al., 2012). Obesity causes the development of gastroesophageal reflux disease (GERD) and Barrett’s esophagus, conditions that predispose individuals to carcinogenesis (Schlottmann et al., 2020). The accumulation of abdominal fat increases intra-abdominal pressure, which exerts a mechanical effect on gastric pressure, disrupting the transdiaphragmatic pressure gradient and promoting the development of GERD (El-Serag et al., 2006; De Mello Del Grande et al., 2020). A metaplastic transformation of the squamous mucosa of the esophageal epithelium in the distal esophagus with metaplastic columnar epithelium is a feature of Barrett’s esophagus, which is also characterized by increased cell proliferation and reduced apoptosis (Reid et al., 1993; Jung et al., 2011). A meta-analysis demonstrated a strong association between central adiposity and an elevated risk of Barrett’s esophagus, highlighting the link between obesity and this precancerous condition (Singh et al., 2013).

Prostate cancer is the second most prevalent cancer among men in the world, and obesity may accelerate its progression, because higher mortality rates are registered among those diagnosed with this disease (Sung et al., 2021; Haque et al., 2014). While a meta-analysis found no direct link between obesity and the incidence of prostate cancer, it did reveal an increased risk of aggressiveness in obese individuals (Zhang et al., 2015). Reduced prostate-specific antigen (PSA) levels in obese men may contribute to this connection by decreasing the chance of early prostate cancer detection (Vidal et al., 2014). Obesity also disrupts the insulin/IGF-1 axis, alters sex hormone levels, changes adipokine signalling, and heightens oxidative stress, all of which contribute to prostate cancer progression (Bandini et al., 2017). Hyperinsulinemia, caused by obesity, increases IGF-1 secretion, which stimulates angiogenesis, mitogenesis, and inhibits apoptosis, processes that collectively facilitate prostate cancer progression (Bandini et al., 2017; Gallagher and LeRoith, 2010). Obesity also elevates leptin levels, which activate the PI3k/Akt pathway, driving the proliferation, migration, and invasion of prostate cancer cells (Olivas and Price, 2021; Noda et al., 2015).

Glioblastoma is the most prevalent and aggressive malignant brain tumor in adults (Koshy et al., 2012). Although its association with obesity is not yet fully understood, some studies suggest that obesity may correlate with a poorer prognosis in patients with glioblastoma (Chambless et al., 2012). Chronic low-grade inflammation, a hallmark of obesity, characterized by the activation of inflammatory cells and the secretion of inflammatory mediators promotes glioblastoma proliferation, angiogenesis, and invasion (Sowers et al., 2014; Fossati et al., 1999; Murat et al., 2009). In certain glioblastoma cell lines, leptin - an adipokine whose levels are elevated in obesity has been found to activate several intracellular signaling pathways, including NF-κB, p38-MAPK, and JAK/STAT3, all of which drive increased proliferation, migration and invasion (Panza et al., 2020; Yeh et al., 2009). Furthermore, elevated levels of IGF-1, commonly observed in obesity, bind to its receptor, triggering the PI3K/AKT pathway, which plays a critical role in glioblastoma progression by promoting cell proliferation and migration (Tirrò et al., 2020; Hägerstrand et al., 2010; Schlenska-Lange et al., 2008). Because there are so few scientific studies on this subject, more research is needed to clarify the underlying mechanisms and potential therapeutic implications of this association.

Surgery, chemotherapy, radiation therapy, immunotherapy, hormone therapy, and targeted therapy are frequently used in the treatment of cancer (Baudino T, 2015). However, each strategy has limitations. Surgery is only effective when the disease is detected at an early stage, radiation therapy may damage healthy cells and chemotherapy often leads to drug resistance as cancer cells become unresponsive to previously successful treatments (Kaur et al., 2023). These challenges show how urgently new therapeutic approaches are needed to overcome these limitations. Natural compounds have recently been investigated for use in combination with radiotherapy, because those compounds have antioxidant and pro-immune properties, favourable safety profile, potential as radiosensitizer and capacity to protect normal tissues (Nisar et al., 2022).

## 3 Betulinic acid as a treatment for obesity-related disorders

BA, also known as 3-beta-hydroxy-lup20(29)-en-28-oic acid is sourced from the bark of the birch tree (*Betula* sp.), sycamore, eucalyptus and the plane tree (*Platanus* sp.), or it can be synthesized via chemical or enzymatic oxidation of betulin (Csuk, 2014).

BA is known for its multiple biological properties including antiviral, anti-bacterial, anti-cancer, and anti-inflammatory properties, and has been recently suggested as a way to reduce the accumulation of abdominal fat (De Melo et al., 2009; Kim et al., 2019; Pavlova et al., 2003; Schühly et al., 1999; Fulda and Debatin, 2000).

A characteristic of obesity is the presence of low-grade chronic inflammation, which is especially evident through elevated levels of systemic inflammatory markers (Karczewski et al., 2018). When inflammation becomes uncontrolled, it can contribute to several health conditions, such as autoimmune disorders, asthma, inflammatory bowel disease, and cardiovascular issues (Oliveira-Costa et al., 2022). BA inhibits the production of NO, an inflammatory mediator that contributes to certain inflammatory diseases, and phospholipase A2 activity, underscoring its role as an anti-inflammatory agent (Boparai et al., 2017; Yun et al., 2003; Bernard et al., 2001). Furthermore, in human peripheral blood mononuclear cells activated by LPS, BA suppresses inflammatory responses by modulating extracellular signal-regulated kinase (ERK), Akt, and NF-kB signalling (Viji et al., 2011). Therefore, BA emerges as a promising candidate for diminishing the adverse effects of obesity.

### 3.1 Effects of betulinic acid on diabetes

As a metabolic disorder, diabetes is characterized by impairments in insulin production and disruptions in macronutrient metabolism, including carbohydrates, lipids, and proteins (Boparai et al., 2017; Riya et al., 2014). *In vivo* studies show that BA modulates key metabolic pathways involved in glucose absorption and uptake, endogenous glucose production and insulin sensitivity and resistance, making it beneficial for the treatment of T2DM (De Melo et al., 2009; Ríos and Máñez, 2018; Castro et al., 2014; Kim SJ. et al., 2014).

*In vitro* experiments show that BA effectively reduces *α*-amylase and *α*-glucosidase activity (Kumar et al., 2013). Thus, reducing the intestinal absorption of glucose by enzymes responsible for polysaccharides hydrolysis, there a decreasedrelease of free glucose into circulation (Ríos and Máñez, 2018; Kumar et al., 2013). Furthermore, treatment of β-pancreatic cells with BA induces calcium influx through a multifaceted mechanism involving ATP-dependent potassium channels and calcium-dependent chloride channels, collectively contributing to its stimulation of insulin secretion (Esievo et al., 2024; Castro et al., 2018). Additionally, using a high-fat diet-fed mice model, BA orally administered promotes glycogen synthesis by activation of the p-adenosine 5′-monophosphate-activated protein kinase (AMPK) pathway, which suppresses the expression of key gluconeogenic enzymes, such as phosphoenolpyruvate carboxykinase (PEPCK) and glucose-6-phosphatase (G6Pase), thereby decreasing hepatic production of glucose (Ríos and Máñez, 2018; Kim SJ. et al., 2014).

Numerous experimental studies have explored the beneficial effect of BA on diabetes. Xie et al. reported that BA was intragastrically administered to diabetic Sprague–Dawley rats, which significantly reduced blood glucose and insulin levels, suggesting its protective role in managing diabetes (Xie et al., 2017). Similarly, Salau et al. proved in an *in vitro* study that BA inhibited the activity of *α*-amylase and α-glucosidase (Salau et al., 2024). Furthermore, Kim, et al. observed that gene expression of gluconeogenic proteins (PEPCK and G6Pase) was inhibited by BA treatment in HepG2 cells, thereby reducing hepatic glucose production (Kim SJ. et al., 2014). Moreover, findings from these studies also revealed that BA increased the phosphorylation of AMPK in HepG2 cells, indicating AMPK activation as an important pathway in the suppressive effect of BA (Kim SJ. et al., 2014).

Despite its promising antidiabetic effects, a study by Ahangarpour, et al. reported adverse outcomes. They observed that oral administration of BA via gavage for 2 weeks in STZ–nicotinamide diabetic mice induced adverse reproductive effects, such as lower testosterone, reduced sperm counts, and histopathological deterioration of the seminiferous tubules (Ahangarpour et al., 2016). Most research on BA’s anti-diabetic effects has been conducted in rodent models, which may not fully translate to the complex human pathophysiology of T2DM. In addition, evidence also showed that very high doses of BA (2,000 mg/kg) caused temporary anaemia in rats. Although the effect was reversed later, it suggests that high doses of BA could worsen anaemia in diabetic patients, highlighting the importance of cautious dosing and further safety testing (Esievo et al., 2023).

### 3.2 Effects of betulinic acid on cardiovascular diseases

BA has gained attention as a potential therapeutic agent for managing CVD due to its ability to diminish the progression of atherosclerotic lesions and myocardial dysfunction (Silva et al., 2016). Atherosclerosis begins with vascular inflammation, driven by damage from atherosclerotic plaques, which may contribute to the development of cardiovascular complications (Yoon et al., 2017; Choi et al., 2003) *In vitro* studies was showed that,BA counteracts this process by suppressing NF-κB activation and ROS production, which reduces inflammatory cells recruitment (Yoon et al., 2010). BA also improves endothelial function by decreasing expression of adhesion molecules like ICAM-1, VCAM-1, E-selectin, and endothelin-1 (ET-1), as demonstrated in apolipoprotein E knockout (ApoE KO) mice administrated oral BA, all of which are implicated in the progression of coronary atherosclerosis (Yoon et al., 2017). Additionally, treatment of BA in endothelial cells promotes vasorelaxation by enhancing NO production via endothelial nitric oxide synthase (eNOS) activation, driven by the AMPK pathway (Yoon et al., 2017; Jin et al., 2016). Some examples demonstrating BA’s effects on CVD include a study by Afghan, et al. showing that a high-fat diet in BALB/c mice led to elevated levels of total cholesterol, triglycerides, and LDL, indicative of atherosclerotic changes (Afghan et al., 2022). This investigation demonstrated that BA was administrated orally and effective in reducing total cholesterol, LDL, VLDL, and triglyceride levels while increasing HDL levels in BALB/c mice, underscoring the protective role of BA against CVD (Afghan et al., 2022). Other case is the study report by, Yoon, et al., in which orally administeredBA was found to reduce atherosclerotic lesions and to prevent the decrease of eNOS expression in ApoE gene-deficient C57BL6J mice, further supporting its anti-atherosclerotic properties (Yoon et al., 2017). Despite the potential positive properties of BA, several limitations restrict its use in cardiovascular disease treatment. BA has very low oral bioavailability, with less than 1% of the administered dose absorbed into circulation, which limits its therapeutic effectiveness (Godugu et al., 2014). Furthermore, clinical trials directly evaluating BA in cardiovascular patients are lacking, so its safety profile in this context remains uncertain (Silva et al., 2016). While BA has not been directly linked to cardiovascular toxicity, the absence of human studies and the possibility of adverse effects at higher doses raise important safety concerns for its use in cardiovascular therapy.

### 3.3 Effects of betulinic acid on nonalcoholic fatty liver disease

BA also has the potential to be a therapeutic agent for NAFLD. The compound works as an agonist of the farnesoid X receptor (FXR), which is a critical regulator of lipid and glucose metabolism, inflammatory responses, and the liver’s energy homeostasis (Yao and Liu, 2022; Gu et al., 2019). Through FXR activation, BA mitigates hepatic ER stress in vitroby suppressing the PERK/EIF2α/ATF4 signalling pathway, which is known to contribute to hepatic steatosis (Gu et al., 2019; Wang et al., 2023). FXR activation also decreases lipotoxicity, promotes cholesterol metabolism, improves insulin resistance, and exerts anti-inflammatory effects (Gu et al., 2019). Furthermore, BA regulates fat metabolism by reducing lipid accumulation and fat synthesis, while stimulating fatty acid oxidation, in human hepatocyte-derived cells (Quan et al., 2013). Additionally, Mu, et al. reported that BA, administered either by oral gavage or incorporated into a high-fat diet, suppresses the activity of fatty acid synthase (FAS), by downregulating the expression of Yin Yang 1 (YY1), a key upstream regulator of FAS (Mu et al., 2020). BA has also been shown to attenuate liver fibrosis and represents a promising candidate for the development of anti-fibrotic therapies aimed at preventing NASH (Mu et al., 2020).

Some studies have explored the association between BA and NAFLD. Gu, et al. demonstrated that BA acts as a selective activator of FXR in HEK293T cells and that C57BL/6J diet-induced obesity mice, receiving BA mixed into their high-fat diet, exhibited lower levels of hepatic steatosis, as evidenced by a decrease in hepatic lipid content, along with decreased expression of hepatic ER stress-related genes, indicating BA’s ability to attenuate ER stress during hepatic steatosis (Gu et al., 2019). Moreover, they analysed liver damage markers, such as ALT and AST, finding that BA treatment significantly lowered ALT levels but not AST levels, suggesting an improvement in hepatic steatosis in C57BL/6J diet-induced obesity mice (Gu et al., 2019). Furthermore, Zhao et al. investigated the anti-fibrotic effects of BA and proved that C57BL/6J mice with hepatic fibrosis, treated daily with BA by oral gavage, exhibited lower collagen amounts in liver tissues compared to untreated controls, confirming that BA attenuates liver fibrosis (Zhao et al., 2022).

Although BA shows promising effects in NAFLD models, current evidence is primarily preclinical, and there are no direct reports of toxicity in these studies. However, potential risks at higher doses cannot be ruled out, and some data from other models suggest dose-dependent cytotoxicity in non-target cells (Yao and Liu, 2022). Moreover, gaps remain regarding its pharmacokinetics, long-term safety, and possible off-target effects in humans. Therefore, clinical studies are needed to confirm both the efficacy and safety of BA before it can be recommended as a therapy for NAFLD.

### 3.4 Effects of betulinic acid on cancer

The anti-cancer effect of BA rises essentially from its capacity to induce apoptosis. Apoptosis, or programmed cell death, proceeds via two main routes: the intrinsic or mitochondrial pathway and the extrinsic or death receptor pathway (Kiraz et al., 2016). The mitochondrial pathway is regulated by anti-apoptotic proteins, such as members of the Bcl-2 family, and pro-apoptotic proteins like Bax (Mullauer et al., 2010). *In vitro* studies demonstrate that BA promotes the mitochondrial pathway, promoting the permeabilization of the outer mitochondrial membrane (MOMP), which results in the release of cytochrome c and Smac into the cytosol, ultimately triggering the activation of cytosolic caspase (Fulda, 2008; Kumar et al., 2018; Wang et al., 2017). Furthermore, BA enhances the expression of proapoptotic proteins while suppressing antiapoptotic proteins in colorectal cancer cells, which promotes apoptosis (Zeng et al., 2019).

**FIGURE 3:**
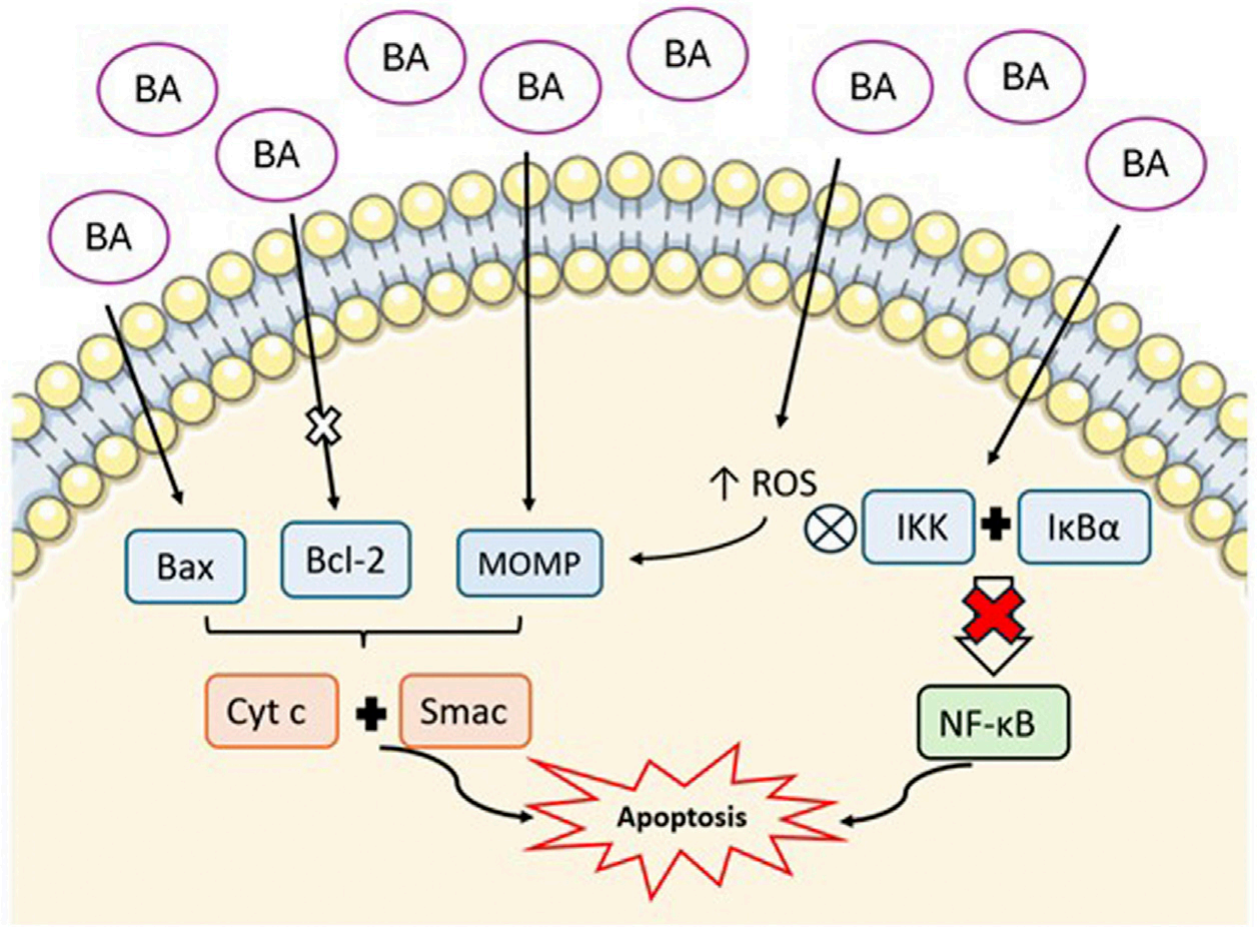
Representation of the mechanism of BA-induced apoptosis via mitochondrial pathway. BA increases pro-apoptotic proteins (Bax), inhibits anti-apoptotic proteins (Bcl-2), and promotes mitochondrial outer membrane permeabilization (MOMP). This results in the release of cytochrome c (Cyt c) and Smac into the cytosol. Additionally, BA increases reactive oxygen species (ROS) and inhibits NF-κB pathway by supressing IKK complex and IκBα. All these mechanisms culminate in the promotion of apoptosis. (*Image created with an icon from* smart.servier.com).

In cancer cells apoptosis induced by BA occurs through the increase in ROS production, a process that is also elevated in obesity, and contributes to the initiation of MOMP (Kumar et al., 2018; Csuk et al., 2010). Additionally, the activation of the NF-κB pathway is a characteristic of many cancer types, driving epigenetic changes, EMT, angiogenesis, metastasis, drug resistance, and immunosuppression (Jiang et al., 2021). The phosphorylation of IκB proteins is driven by increased activity of the inhibitor of nuclear factor-κB kinase (IKK) followed by the degradation of the IKK complex itself (Jiang et al., 2021; Shankar et al., 2017). This releases NF-κB dimers, which translocate to the nucleus, where p65 subunit activates the NF-κB, binding to specific DNA sequences and upregulating target genes involved in proliferation and anti-apoptotic signalling (Jiang et al., 2021; Shankar et al., 2017). *In vitro* studies have shown that BA inhibits these effects by reducing nuclear levels of NF-κB/p65, inhibiting IKK activity, and stimulating IκBα phosphorylation at serine 32/36, leading to its degradation (Lou et al., 2021; Rabi et al., 2008). Therefore, the inhibition of the NF‐κB signaling pathway by BA, induces apoptosis and the suppression of cell proliferation (Su et al., 2017). These mechanisms are represented in Figure 3.

BA’s anti-cancer effect may also rely on the inhibition of angiogenesis and metastasis. BA has been shown to reduce the expression of Sp transcription factors (Sp1, Sp3, Sp4) in breast cancer cells, suppressing VEGF and its receptor (VEGFR), which prevents new blood vessel formation (Luo et al., 2016; Yang et al., 2014). In addition, BA suppresses HIF-1α levels by inhibiting prolidase (an enzyme central to angiogenic signalling) andreduces integrins expression in endometrial cancer cells, inhibits aminopeptidase *N* activity *in vitro* and suppresses signal transducer and activator of transcription 3 (STAT3) expression in prostate cancer cells (Karna et al., 2010; Shin et al., 2011; Melzig and Bormann, 1998). In what concerns anti-metastatic activity, BA reduces the expression of matrix metalloproteinase MMP-2 and MMP-9 while increases tissue inhibitor of metalloproteinase 2 (TIMP2) in breast cancer cells and E-cadherin levels in gastric cancer cells (Amiri et al., 2020; Zeng et al., 2017; Chen et al., 2020).

Evidence shows that BA is effective in the treatment of many cancers associated with obesity, such as breast cancer, prostate cancer, colorectal cancer, endometrial cancer, glioblastoma and many others (Ali-Seyed et al., 2016; Tiwari et al., 2014; Chintharlapalli et al., 2007; Aisha et al., 2012; Karna and Palka, 2009; Bache et al., 2011).

In breast cancer, BA leads to the arrest of the cell cycle on the G2/M phase and promotes cell apoptosis through the mitochondrial pathway in MCF-7 cells by downregulating the level of expression of Bcl-2 while upregulating the level of expression of Bax (Sun et al., 2013). In addition, BA inhibited angiogenesis in MDA-MB-453 and BT474 cells by repressing the levels of Sp1, Sp3, and Sp4 via disruption of microRNA-27a (Liu et al., 2012). Weber, et al. observed that BA inhibited NF-κB activation and downregulated TNF-α, STAT3, and i-NOS expression in MDA-MB-231 and MDA-MB-468 cells, demonstrating that BA has anti-inflammatory effects (Weber et al., 2014). Finally, BA exerts also anti-metastatic activity by reducing MMP-2 and MMP-9 secretion while at the same time upregulating TIMP2 in 4T1 and MDA-MB-231 cells (Zeng et al., 2017).

BA has shown potential as a therapeutic agent for prostate cancer, as it affects selectively cancer cells and leaves the normal cells unharmed (Reiner et al., 2013). In prostate cancer cell lines LNCaP and PC3, BA promotes the degradation of pro-survival proteins, including the androgen receptor (AR), which is significant because AR signalling strongly influences the progression of prostate cancer, while normal cells remain unaffected (Reiner et al., 2013). Shankar et al. demonstrated that BA suppressed cell viability and stimulated apoptosis in LNCaP and DU145 cells (Shankar et al., 2017). Key mechanisms driving cancer progression include dysregulated NF-κB activity and p53 inactivation, which is involved in programmed cell death responses to DNA damage in many cancer cells (Shankar et al., 2017; Schneider et al., 2010; Murphy et al., 2011). Additionally, the same authors showed that BA inhibited the NF-κB pathway in LNCaP and DU145 cells by reducing phosphorylation of IκBα at Ser32/36, thereby enhancing its stability and preventing the nuclear translocation and DNA binding activity of NF-κB/p65, ultimately triggering apoptosis (Shankar et al., 2017).

The impact of BA on colorectal cancer has also been extensively studied. Chintharlapalli, et al. found out that BA inhibits the proliferation of cancer cells by promoting the suppression of Sp1, Sp3, and Sp4 in SW480 and RKO colorectal cancer cell lines (Chintharlapalli et al., 2011). Zeng et al. further observed that BA decreases cell growth in HCT116 cells by downregulating BCL-2, upregulating Bax, and disrupting mitochondrial membrane potential, while simultaneously increasing ROS production (Zeng et al., 2019). These findings suggest that apoptosis induced by BA in colorectal cancer cells may be mediated by mitochondria (Zeng et al., 2019). Additionally, research carried out by Su, et al. showed that BA also reduces proliferation and induces apoptosis in HT-29 cells (Su et al., 2017). Furthermore, Zeng, et al. also revealed that BA-treated HCT116 cells showed repressed migration and invasion ability. This was confirmed by significantly altered levels of MMP-2 and MMP-9 expression, along with reduced TIMP-2 levels (Zeng et al., 2019).

Some studies have looked at the role of BA on endometrial cancer. Karna and Palka showed that BA was able to induce a 50% reduction in viability of Ishikawa endometrial cancer cell line (Karna and Palka, 2009). Furthermore, Karna, et al. demonstrated that BA reduced both collagen production and prolidase activity, an enzyme involved in collagen degradation and angiogenic signalling pathways, in Ishikawa cells (Karna et al., 2010). Additionally, Ishikawa cells treated with BA expressed decreased levels of HIF-1α, which is a key factor in angiogenesis, and VEGF (Karna et al., 2010). Furthermore, BA downregulated the expression of α1 and α2 integrin subunits, thus indicating that BA can interfere with the angiogenic process through HIF-1α, VEGF, and integrin signaling pathways in these cells (Karna et al., 2010).

Beyond the antitumor effects of BA on epithelial cancers, it has been shown to induce apoptosis in non-epithelial cancers (Fulda et al., 1999). Some examples are glioblastoma and neuroectodermal tumour cells, where activation of the caspase cascade has an important role (Fulda et al., 1999; Fulda et al., 1997). In glioblastoma, the antitumour role of BA has been extensively studied, particularly its impact on cell proliferation, migration, and apoptosis (Fernandes et al., 2024; Lo et al., 2020). Yaozu et al. observed that BA reduced the proliferative potential of glioblastoma cell lines A172 and U87MG while promoting apoptosis (Yaozu et al., 2020). This study further revealed that BA inhibited NF-κB activation, by decreasing intracellular levels of NF-κB p65 in A172 and U87MG cell lines, reduced BCL-2 levels suppressed the levels of the X-linked inhibitor of apoptosis protein (XIAP), and increased the expression of pro-apoptotic markers such as BAX, caspase-3, and caspase-9, indicating that BA promotes apoptosis by suppressing the NF-κB signalling pathway (Yaozu et al., 2020). Moreover, Wei-Lun, et al. showed that BA enhances the therapeutic efficacy of temozolomide (TMZ) in glioblastoma, particularly in TMZ-resistant cells (Lo et al., 2020). TMZ, normally used for glioblastoma treatment, combined with BA was shown to inhibit glioblastoma progression, with BA suppressing the expression of Sp1 indicating a potential combined therapeutic strategy (Lo et al., 2020). In contrast, the impact of BA on angiogenesis and metastasis in glioblastoma has not been fully explored, highlighting the need for further investigations into these mechanisms, particularly its effects on VEGF, MMP-2, and MMP-9.

Obesity is also know to contribute to the progression of melanoma, and the potential therapeutic role of betulinic acid (BA) in this context has also been investigated (Dobbins et al., 2013). Coricovac et al. showed thst BA reduced the viability of A375 human melanoma cells by inducing mitochondrial dysfunction and promoting apoptosis (Coricovac et al., 2021). Similarly, Liu et al. reported that BA increases ROS production and disrupts mitochondrial membrane potential in B16 murine melanoma cells, resuting in apoptosis (Liu et al., 2004). Expending on these findings, Tan et al. showed that BA induces apoptosis in UISO-Mel-1 melanoma cells through the activation of pro-apoptotic MAPK signaling pathways, particularly p38 and SAP/JNK (Tan et al., 2003). In another study, BA significantly reduced the proliferation of ME20, ME21, A375, 518A2 melanoma cells and and exhibited an additive effect when combined with radiotherapy (Selzer et al., 2000). Furthermore, Gheorgheosu et al. demonstrated that BA suppresses EMT in A375 melanoma cells by downregulating mesenchymal markers and upregulating epithelial markers, highlighting its potential to counteract melanoma metastasis (Gheorgheosu et al., 2013).

The therapeutic impact of BA on lung cancer, which can be exacerbated by central obesity, has also been investigated (Gao et al., 2019). Hsu et al. demonstrated that BA inhibits the transcription factor Sp1 in A549 and H1299 lung cancer cells, leading to reduced expression of cyclin A2, resulting in cell cycle arrest at the G2/M phase, and ultimately inhibition of cell proliferation (Hsu et al., 2012). Additionally, Zuco et al. reported that BA effectively reduces the proliferation of H460 and POGB lung cancer cells while exhibiting significantly lower toxicity toward normal cells, highlighting its potential as a safer anticancer agent (Zuco et al., 2002). Kessler et al. further observed that BA induced significant cell death in the A549, H187, N417, and MBA9812 lung cancer cell lines, with viability dropping below 10% after 48 h of treatment, suggesting strong cytotoxic effects in both non-small and small cell lung cancer types (Kessler et al., 2007). Furthermore, another study reported that BA induces apoptosis in paclitaxel-resistant H460 lung cancer cells by reducing cell viability, disrupting mitochondrial membrane potential, and increasing the Bax/Bcl-2 ratio, which highlights its potential to overcome chemoresistance (Zhan et al., 2018).

BA has demonstrated significant effects on pancreatic cancer growth, a malignancy closely linked to obesity (Genkinger et al., 2011). Guo et al. studied the effect of BA on PANC-1 and SW1990 pancreatic cancer cell lines and observed that BA reduced cell viability in a dose-dependent manner and effectively induced apoptosis through activation of the AMPK/mTOR signaling pathway (Guo et al., 2020). Additionally, the study by Sun et al. showed that BA suppresses this migration and invasion of Mia PaCa-2 and PANC-1 cells by inhibiting EMT, as evidenced by increased E-cadherin expression and reduction of vimentin levels (Sun et al., 2018). In another study, BA was also shown to significantly inhibit the viability of Mia PaCa-2 and SUIT-2 pancreatic cancer cells, at low concentrations, while sparing normal pancreatic cells, which suggests its therapeutic imapact in pancratic cancer (Chiu et al., 2021). This study further showed that BA upregulates genes involved in metabolism and tumor suppression, while downregulating genes associated with inflammation and cell migration, suggesting a role in reducing the migratory capacity of PDAC cells (Chiu et al., 2021).

Obesity increases the risk of ovarian cancer, a disease in which the effects of BA have also been investigated (Bander et al., 2016). In the study by Lee et al., BA reduced the viability of A2780 human ovarian carcinoma cells in a dose-dependent manner, demosntrating significant cytotoxic effects (Lee et al., 2019). Additionally, BA induced apoptosis, as evidence by incresed activation of caspases-3, -8, and -9, upregulation of Bax, and downregulation of Bcl-2, indicating involvement of both mitochondrial and death receptor pathways (Lee et al., 2019). In another study by Liao et al., BA also suppressed the proliferation of SKOV3 and SW626 ovarian cancer cells, while exhibiting minimal cytotoxicity toward normal cells (Liao et al., 2020). Furthermore, BA significantly inhibited cell migration and invasion by blocking EMT, as shown by decreased N-cadherin and increased E-cadherin expression (Liao et al., 2020). *In vivo*, intraperitoneal administration of BA reduced tumor volume and weight in a SKOV3 xenograft mouse model and downregulated Ki-67 and MMP-2 expression, supporting its anti-metastatic and anti-proliferative activity (Liao et al., 2020).

All these findings prove BA’s efficacy as a therapeutic agent for several obesity-related disorders. The summary of BA effects on those diseases is presented in Table 1.

**TABLE 1:** Summary of BA’s effect on obesity-related disorders.

Obesity-related disorder	*In vitro*/*in vivo* models	Effects of BA	References
Diabetes	HepG2 cells Sprague-Dawley rats	↓ Glucose levels ↓ Insulin levels ⦻ *α*-amylase ⦻ α-glucosidase ⦻ gluconeogenic proteins ↑ AMPK activation	Kim et al. (2014a) Xie et al. (2017) Salau et al. (2024)
Cardiovascular diseases	BALB/c mice C57BL6J mice	↓ total cholesterol levels ↓ LDL levels ↓ VLDL levels ↓ triglycerides levels ↑ HDL levels ↓ atherosclerotic lesions	Yoon et al. (2017) Afghan et al. (2022)
NAFLD	HEK293T cells C57BL/6J mice	↓ Hepatic steatosis ↑ Hepatic lipid content ↓ ALT levels ↓Hepatic ER stress ↓ Liver fibrosis	Gu et al. (2019) Zhao et al. (2022)
Breast Cancer	MCF-7 cells MDA-MB-468 cells MDA-MB-231cells BT474 cells 4T1 cells	↑ Apoptosis ⦻ Angiogenesis ⦻ Inflammation ⦻ Metastasis	Sun et al. (2013) Liu et al. (2012) Weber et al. (2014) Zeng et al. (2017)
Prostate Cancer	LNCaP cells PC3 cells DU145 cells	Degradation of AR ↓ Cell viability ⦻ NF-κB pathway ↑ Apoptosis	Reiner et al. (2013) Shankar et al. (2017) Schneider et al. (2010) Murphy et al. (2011)
Colorectal Cancer	SW480 cells RKO cells HCT116 cells HT-29 cells	⦻ Cell proliferation ↓ Cell growth ↑ Apoptosis ⦻ Migration and invasion	Zeng et al. (2019) Su et al. (2017) Chintharlapalli et al. (2011)
Endometrial Cancer	Ishikawa cells	↓ Cell viability ↓ Collagen production ↓ Prolidase activity ⦻ Angiogenesis	Karna and Palka (2009) Karna et al. (2010)
Glioblastoma	A172 cells U87MG cells Balb/c mice Sprague-Dawley rats	↑ Apoptosis ⦻ NF-κB activation ↓ Bcl-2 ↑ Bax, caspase-3, caspase 9 ⦻ Sp1 Penetrates BBB	Lo et al. (2020) Yaozu et al. (2020) Killinger et al. (2014) Li et al. (2022)
Melanoma	A375 cells B16 cells UISO-Mel-1 cells ME20 cells ME21 cells 518A2 cells	↓ Cell viability ↑ Apoptosis ↑ ROS production ↓ Cell proliferation ⦻ EMT	Dobbins et al. (2013) Coricovac et al. (2021) Liu et al. (2004) Tan et al. (2003) Selzer et al. (2000) Gheorgheosu et al. (2013)
Lung Cancer	A549 cells H1299 cells H460 cells POGB cells H187 cells N417 cells MBA9812 cells	⦻ Sp1 ↓ Cell proliferation ↑ Cell death ↓ Cell viability ↑ Apoptosis ↑ Bax/Bcl-2	Hsu et al. (2012) Zuco et al. (2002) Kessler et al. (2007) Zhan et al. (2018)
Pancreatic Cancer	PANC-1 cells SW1990 cells Mia PaCa-2 cells SUIT-2 cells	↓ Cell viability ↑ Apoptosis ⦻ Migration and invasion ⦻ EMT ↑ E-cadherin ↓ Vimentin ↑ Genes involved in metabolism and tumor suppression ↓ Genes associated with inflammation and cell migration	Guo et al. (2020) Sun et al. (2018) Chiu et al. (2021)
Ovarian Cancer	A2780 cells SKOV3 cells SW626 cells SKOV3 xenograft mouse	↓ Cell viability ↑ Apoptosis ↑ caspase 3, 8, 9 ↑ Bax ↓ Bcl-2 ↓ Cell proliferation ⦻ Migration and invasion ⦻ EMT ↓ Tumor volume and weight ↓ Ki-67 ↓ MMP-2	Lee et al. (2019) Liao et al. (2020)

⦻ - inhibition; ↑ - increase; ↓ - reduction.

Regarding the effect of BA on cancer, some contraditory information has been reported. Although BA often inhibits NF-κB to suppress cancer cell proliferation, it can also activate NF-κB in a cell-type-specific manner (Fulda, 2008). In some cancer cells, this activation contributes to BA-induced apoptosis, as blocking NF-κB reduces apoptotic effects (Fulda, 2008). These findings indicate that BA’s impact on NF-κB is context-dependent, highlighting the complexity of its anticancer mechanisms. Although BA is generally reported to spare normal cells, several studies indicate that at high concentrations or prolonged exposure, BA can induce cytotoxicity in normal cells. For example, murine and human fibroblasts can exhibit reduced viability and mitochondrial dysfunction at BA doses near those effective in cancer cells, indicating context-dependent selectivity (Małaczewska et al., 2021).

### 3.5 Challenges and limitations in the therapeutic use of betulinic acid

BA displays poor pharmacokinetic properties, largely due to its extremely low aqueous solubility, which limits gastrointestinal absorption to less than 1%, resulting in minimal systemic bioavailability (Jiang et al., 2021; Jäger et al., 2009). Experimental studies in rodents confirm high plasma protein binding and uneven tissue distribution, with substantial accumulation in organs such as lymph nodes, ovaries, and liver, while circulating levels remain consistently low. In mice, BA intraperitoneally administered at doses of 250 or 500 mg/kg had elimination half-lives of 11.5 and 11.8 h, and total clearances of 13.6 and 13.5 L/kg/h, where serum concentrations peaking at 0.15 and 0.23 h, respectively (Ríos and Máñez, 2018; Jäger et al., 2009). These limitations complicate both *in vitro* and *in vivo* experimental designs and restrict the compound’s therapeutic potential. To address these issues, researchers have explored different strategies, including polymer complexes, mucoadhesive microparticles, nanocarriers, and structural modifications. Although these approaches have improved solubility and significantly enhanced oral bioavailability in experimental models, they remain at a preclinical stage (Ríos and Máñez, 2018).

Metabolic studies further show that BA undergoes transformation through human CYP enzymes, with C-28, C-6, and C-23 being the main sites of oxidative metabolism. While chemical modifications at specific positions have been shown to improve solubility without compromising activity, novel delivery strategies such as liposomes, nanoemulsions, transdermal systems, and sustained-release formulations are also under investigation (Godugu et al., 2014; Wang et al., 2024).

Even though BA demonstrates considerable therapeutic potential for several diseases, this compound has other several limitations. A central problem is BA’s extraction and production, because the direct extraction method produces lower quantities and chemical synthesis demands strict reaction conditions (Jiang et al., 2021; Jäger et al., 2009). For example, extraction with 70% ethanol yielded 23.76 mg of BA per 10 g of birch bark, whereas ultrasonic extraction produced only 0.0021% (Mukherjee et al., 2010; Kim H-I. et al., 2014). Particularly, regarding brain cancers treatment, the cross of the blood-brain barrier (BBB) is still an obstacle for therapeutic agents. This happens because of the presence of tight junctions and active transport mechanisms and the selectivity of this barrier (Fernandes et al., 2024). Interestingly, BA can cross the BBB, supressing one of the primary obstacles to treating glioblastoma and other neurological disorders (Killinger et al., 2014).

One promising strategy to address BA’s limited access to brain tissue is the development of BA micro or nanoparticles, as is the case of hybrid polymer nanoformulations with targeted ligands, which enhances its stability, sustained realease and safety (Li et al., 2022). Their biochemical versatility allows them to integrate multiple properties from different materials into a single micro or nanoformulation, enhancing their therapeutic potential (Roque et al., 2023). In preclinical studies, nanoparticles successfully penetrated the brains of mice, reducing ischemia-induced infarction and demonstrating significant antitumoral effects in glioblastoma models (Li et al., 2022; Deng et al., 2019). Despite promising advances, the development of effective administration routes and stable formulations remains one of the greatest challenges to translating BA into clinical application.

Another important approach is the use of ionic liquid formulations, which increase the aqueous solubility of BA and improve its therapeutic action (Suresh et al., 2012). In fact, some studies have demonstrated that synthesized organic salts and BA ionic derivatives exhibit stronger antiproliferative activity and higher cytotoxicity against various cancer cell lines (Suresh et al., 2012; Silva et al., 2019). Furthermore, in another study, novel indole-functionalized BA derivatives have exhibited high cytotoxicity against several cancer cell lines, indicating their potential as promising candidates for cancer treatment (Fernandes et al., 2024; Bębenek et al., 2022).

Regarding BA’s toxicity, evidence suggests that spray-dried (SD) formulations of BA, caused no changes in body weight in animal models, and histopathology of the intestine, liver, lung, heart, spleen, and kidney showed no tissue damage (Godugu et al., 2014). Furthermore, BA has some adverse effects, including very low oral bioavailability which, together with the scarcity of clinical trials in humans, leaves its efficacy and long-term safety in cardiovascular, metabolic, hepatic, or oncological contexts uncertain. Preclinical studies report dose-dependent cytotoxicity in non-target cells, temporary anaemia at high doses, and reproductive toxicity in diabetic mice. Clinical trials using BA reached phase I/II and almost none of them have progressed to phase III (Ruiz-Molina et al., 2022; Gielecińska et al., 2023). Those studies have often stalled at the animal model testing stage, caused mainly by the lack of evidence concerning the safety of these drugs, potential mid-to long-term toxicity, immunogenicity, and pharmacokinetic profile. Furthermore, BA’s effects on pathways such as NF-κB are context-dependent, showing both inhibitory and activating actions depending on the cell type, which may influence therapeutic outcomes and adverse effects. These results highlight the need for additional preclinical and clinical evaluation to assess the safety and efficacy of BA.

## 4 Main conclusion and future directions

Obesity is a widespread condition that impacts many individuals globally and is associated with an increased risk of several health issues, including diabetes, CVD, NAFLD, and cancer. This review compiles the information on BA use, which shows great therapeutic potential for the management of those obesity-related disorders. Overall, the compound exhibits antioxidant, anti-inflammatory, and antitumoral effects, as well as impacting glucose and lipid metabolism, apoptosis, angiogenesis and metastasis.

Despite BA showing great potential in laboratory studies, clinical results are still in the early stages, where the efficacy, safety and the best protocols of the compound are being explored. Unlocking the full therapeutic potential of BA could open the way for transformative advancements in treating obesity-related diseases, offering hope for millions worldwide.

This work received financial support from the PT national funds (FCT/MECI, Fundação para a Ciência e Tecnologia and Ministério da Educação, Ciência e Inovação) through the project UID/50006 -Laboratório Associado para a Química Verde - Tecnologias e Processos Limpos.
